# Subsurface Drip Irrigation Combined with Ammonium Enhances Root Growth in Rice (*Oryza sativa* L.), Leading to Improved N Uptake and Higher Yield Formation

**DOI:** 10.3390/plants14060891

**Published:** 2025-03-12

**Authors:** Yuman Cui, Weidong Ma, Changnan Yang, Ruxiao Bai, Tianze Xia, Changzhou Wei, Xinjiang Zhang, Guangwei Zhou

**Affiliations:** 1 Department of Agricultural Resources and Environment, College of Agriculture, Shihezi University, North 4th Street No. 221, Shihezi 832003, China; cuiyuman2022@163.com (Y.C.);; 2 Key Lab of Oasis Ecology Agriculture of Xinjiang Production and Construction Group, Shihezi University, North 4th Street No. 221, Shihezi 832003, China; 3 Xinjiang Production & Construction Corps Key Laboratory of Efficient Utilization of Water and Fertilizer, Shihezi 832000, China; 4 Tandon School of Engineering, New York University, Brooklyn, NY 11201, USA; tx717@nyu.edu; 5 Institute of Agricultural Resources and Environment, Xinjiang Academy of Agricultural Sciences, Urumqi 830091, China

**Keywords:** root spatial distribution, root activity, rice, N uptake, yield

## Abstract

Coordinating the spatial distribution of crop roots with soil nutrients, along with selecting appropriate types of fertilizers, is an effective strategy to enhance root nutrient absorption and increase crop yield. In Xinjiang’s current surface drip irrigation practices for rice (*Oryza sativa* L.), premature leaf senescence and N deficiency are common issues, resulting in decreased yields. This study investigated whether different N forms under subsurface drip irrigation can modulate rice root morphological strategies to delay senescence in later growth stages, enhancing rice N uptake and yield formation. A field experiment compared the effects of different drip irrigation positions (surface drip irrigation at the surface, DI0; subsurface drip irrigation at 10 cm depth, DI10) and N forms (urea N, UN; ammonium N, AN) in four combination treatments (DI0-UN, DI0-AN, DI10-UN, DI10-AN) on rice root morphology, aboveground growth, and yield formation. During the grain-filling stage, the total root length (RL) and root number (RN) in the DI10-AN treatment were higher than in other treatments. Root vitality increased by 23.24–133.72% during the later filling stages, while the root decline rate decreased by 1.16–32.80%. The root configuration parameters β in the DI10-AN treatment were superior to those in other treatments, indicating that roots tend to distribute deeper in the soil. The DI10-AN treatment reduced Malondialdehyde (MDA) levels and increased Superoxide Dismutase (SOD) activity, thereby alleviating water and N stress on the leaves in later growth stages and maintaining higher photosynthetic parameter values. The DI10-AN treatment significantly increased N absorption (14.37–52.88%) and yield (13.32–46.31%). Correlation analysis showed that RL, RN, and root activity (Ra) were significantly positively correlated with transpiration rate (Tr), intercellular CO_2_ concentration (Ci), N uptake (NUP), one thousand-kernel weight (TKW), seed setting rate (SR), Efficient panicle (EP), and yield (r > 0.90). This study presents a new rice drip fertigation technique that combines subsurface irrigation with ammonium to enhance root growth and increase crop productivity.

## 1. Introduction

Drip-irrigated rice (*Oryza sativa* L.) has significant potential for conserving water and increasing yields, making it an important agricultural technology for achieving water savings and ensuring food security in arid regions. However, the practical implementation of drip irrigation for rice production often faces challenges, such as early leaf senescence and decreased yields. Research shows that high-frequency surface drip irrigation causes rice roots to tend to be distributed to shallow layers, and the migration of nitrate to deep soil is the main reason for the reduction of rice yield and premature senescence under drip irrigation [1]. This highlights the need for further optimization and refinement of water and fertilizer management practices for drip-irrigated rice.

Roots are vital organs for nutrient and water acquisition in crops. Root morphology, architecture, and activity significantly affect a plant’s capacity to obtain nutrients from the soil [2,3,4]. Plant roots exhibit both morphological and physiological plasticity, which facilitates enhanced resource acquisition in response to spatial nutrient heterogeneity [5,6,7]. High-yielding rice is characterized by large root biomass, high root vitality, and a large proportion of deep roots, which are essential for ensuring that rice obtains more water and nutrient resources to form a high yield [8,9].

The distribution and dynamics of crop roots (root length, production, mortality, and longevity) play a vital role in soil-plant interactions, such as soil nutrient fluxes and water cycling, thereby affecting crop productivity [10,11]. A thorough understanding of the dynamics of roots under varying water and N supply is essential to explore soil-root interactions and quantify plant growth relationships [12,13]. Non-destructive root detection technologies, such as root tubes and minirhizotrons, are extensively used to measure root morphological parameters, fine root elongation rates, and root turnover [14,15]. These techniques overcome the limitations of traditional destructive methods, which cannot differentiate between live and dead roots. The minirhizotron method enables in situ observation of individual root segments without disturbing crop root growth and has been widely utilized in laboratory and field experiments [15,16]. Additionally, plants adjust root growth direction and density to efficiently and sustainably acquire nitrogen (N) resources from deeper soil layers [17]. Therefore, regulating fertilization methods and the targeted application of nutrients can shape root architecture, representing a critical approach to enhancing N-use efficiency in agricultural production [17].

Drip-irrigated rice is grown using deficit irrigation, where soil N primarily exists as inorganic nitrate. Li et al. [18] conducted a study on rice, finding that under water stress, the addition of ammonium increased root hydraulic conductivity, promoted root branching, and enhanced drought tolerance in rice. However, there is limited research on whether ammonium application in drip-irrigated rice can mitigate premature root senescence, particularly through root architectural changes that enhance nutrient acquisition and yield.

Subsurface drip irrigation technology effectively reduces water and nutrient loss due to evaporation, enhances N-use efficiency, and boosts crop yield. Subsurface irrigation promotes deeper root penetration by precisely supplying water, thereby enhancing the crop’s ability to absorb moisture and nutrients from deeper soil layers during later growth stages [19]. Precise management of water and N creates a more stable growth environment for crops, especially in arid or water-scarce conditions, where subsurface irrigation allows potatoes (*Solanum tuberosum* L.) to achieve high yields even in unfavorable climatic conditions [20]. Research indicates that applying N fertilizer at a depth of 10 cm reduces ammonia volatilization, boosts rice growth and grain yield, improves N-use efficiency, and mitigates environmental risks [21]. Increasing the depth of phosphorus fertilization enhances the roots’ efficiency in absorbing N and phosphorus by expanding the absorption area, thereby supplying adequate nutrients for aboveground growth. This process delays leaf senescence post-tasseling, strengthens the leaves’ photosynthetic capacity and antioxidant systems, ensures effective photosynthesis in the later growth stages, and promotes the production of photosynthetic products [22,23]. Currently, the application of subsurface drip irrigation technology has been primarily focused on maize (*Zea mays* L.), with limited research conducted on other crops, such as drip-irrigated rice. Additionally, ammonium exhibits significantly lower mobility in the soil compared to nitrates [24], and rice is classified as an ammonium-preferring plant. Exploring the DI10-AN in rice is essential to determine whether this method enhances the alignment of rice roots with soil N. This approach could help sustain root vitality and prevent premature senescence in drip-irrigated rice.

In this study, drip-irrigated rice served as the experimental material, focusing on the effects of two types of N fertilizers: urea-based N fertilizer (UN) and ammonium-based N fertilizer (AN). Investigating the effects of N application through different irrigation depths (surface drip irrigation and subsurface drip irrigation) on the morphology, structure, and physiological characteristics of rice roots, and elucidating the root traits associated with high rice yields under subsurface drip irrigation, providing both theoretical and practical references for high-yield, efficient rice cultivation. Main hypotheses: (1) DI10-AN optimizes rice root morphology and architecture, reduces root decay rate, and enhances N uptake in the aboveground parts. (2) DI10-AN maintains higher root vitality in later growth stages, decreases malondialdehyde (MDA) levels, increases superoxide dismutase (SOD) activity and photosynthetic rate, thereby reducing water-N stress on leaves and facilitating rice yield formation.

## 2. Materials and Methods

### 2.1. Experimental Site

**Figure 1:**
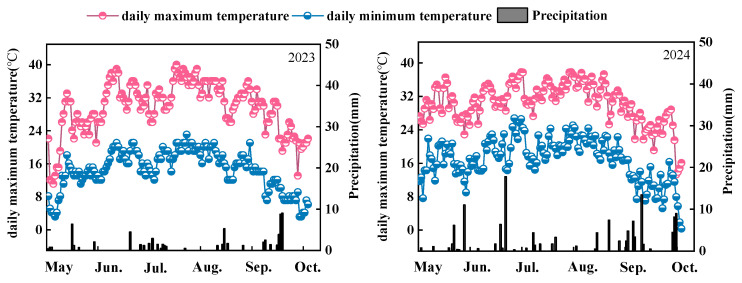
Precipitation and average temperature during the rice-growing season (2023–2024).

Field experiments were conducted from April to October during 2023 and 2024 at the Agricultural Experiment Station, Shihezi University, located in Shihezi City, Xinjiang, China (86°0′39″ E, 44°19′9″ N, altitude 412 m). The region features a temperate continental climate, with an average annual rainfall of 95.7 mm and an average temperature of 22.8 °C during the rice-growing season (Detailed information is shown in Figure 1). The experimental site has been managed under a conventional farming system for many years. The rice-growing season extends from May to September.

### 2.2. Experimental Design and Materials

#### 2.2.1. Experimental Materials

The rice variety used in this experiment was Liangxiang 3 (*Oryza sativa* L.), which is the main cultivar for drip-irrigated rice in the local area.

#### 2.2.2. Experimental Design

The experimental design used a two-factor completely randomized design, with drip irrigation depth and different N forms as factors, and each treatment was replicated three times. Deep N fertilization is an effective method to reduce N loss. Rice roots are primarily concentrated in the tillage layer (0–20 cm), with fewer roots found below this layer [22,25,26,27]. Nutrients required by the plant can be distributed near the root zone, reducing N loss [28,29]. Therefore, four combinations of irrigation and N application treatments were implemented: (1) surface drip irrigation under mulch with conventional urea N (DI0-UN); (2) subsurface drip irrigation under mulch, with pipes buried 10 cm deep, combined with conventional urea N (DI10-UN); (3) surface drip irrigation under mulch with ammonium (DI0-AN); and (4) subsurface drip irrigation under mulch, with pipes buried 10 cm deep, combined with ammonium (DI10-AN). Ammonium N treatments utilized ammonium sulfate (21% N) combined with a nitrification inhibitor (DMPP, at 1% of the N supply) to retard soil nitrification processes. Each plot measures 7 m × 2 m, with three replications per treatment, resulting in a total of 12 treatments. The raw material for urea N is conventional urea (46% N). Total nutrient application during the rice growing season included 300 kg·ha^−1^ of pure N, 110 kg·ha^−1^ of P_2_O_5_, 70 kg·ha^−1^ of K_2_O, 25 kg·ha^−1^ of water-soluble silicon fertilizer, 5 kg·ha^−1^ of boron fertilizer, and 4 kg·ha^−1^ of zinc fertilizer.

The phosphorus (P) fertilizer was monoammonium phosphate (61% P_2_O_5_), and the potassium (K) fertilizer was potassium sulfate (52% K_2_O). N fertilizers were applied in three splits: from emergence to jointing, from jointing to heading, and from heading to maturity, following application ratios of 2:4:4. Rice was cultivated using subsurface drip irrigation under plastic mulch in a dryland farming system, with fertilizers delivered through the irrigation water.

The experiment adopted a 1-film, 2-tube, 4-row planting pattern, with a sowing width of 1.25 m, plant spacing of 10 cm, and a sowing depth of 2–3 cm. Seeds were manually sown, with 6–8 seeds per hole, resulting in a sowing density of 3.0 × 10^5^ holes·ha^−1^. After emergence, rice plants were thinned to 8 per hole at the three-leaf stage. Sowing occurred on 30 April 2023 and 30 April 2024, and seedlings emerged on 10 May 2023 and 8 May 2024. Harvesting took place on 30 September 2023 and 1 October 2024.

The water source for the drip irrigation system is well water, which is either used directly or after undergoing a sun-drying process. During the entire rice-growing period (2023 and 2024), an average of 40 drip irrigation events were conducted, with intervals of 2–4 days and a total amount of 10,980 m^3^·ha^−1^. The irrigation schedule is as follows: 2 events during the sowing period, each applying 300 m^3^·ha^−1^; 10 events during the seedling stage, each applying 300 m^3^·ha^−1^; 10 events during the tillering stage, each applying 280 m^3^·ha^−1^; 5 events during the heading stage, each applying 260 m^3^·ha^−1^; 3 events during the flowering stage, each applying 260 m^3^·ha^−1^; and 10 events during the grain-filling period, each applying 250 m^3^·ha^−1^. The drip irrigation runoff rate was 2.0 h·L^−1^, and soil moisture was monitored using Time Domain Reflectometry (TDR) technology (TRIME-TDR, IMKO, Ettlingen, Germany), which guided irrigation management. Irrigation was initiated when soil moisture content fell below the predetermined threshold for each treatment, and a water meter was installed to measure irrigation volume.

### 2.3. Sample Collection and Processing

#### 2.3.1. Installation of Root Tubes and Collection of Root Parameters

In this experiment, the CI-600 Root System Dynamic Monitoring System was used for targeted sampling at specific points to acquire scanned images. The observation window measured 21.59 cm × 20 cm (0.0439 m^2^). Rootsnap image analysis software (Version 1.4.0.108, Camas, WA, USA), integrated with the root scanner, was employed to process the images and extract relevant data. Fine root classification followed the criteria of Hendrick and Pregitzer (1992), which defined newly observed white roots as new roots and roots that turned either dark brown or black or disappeared as dead roots [30].

**Figure 2:**
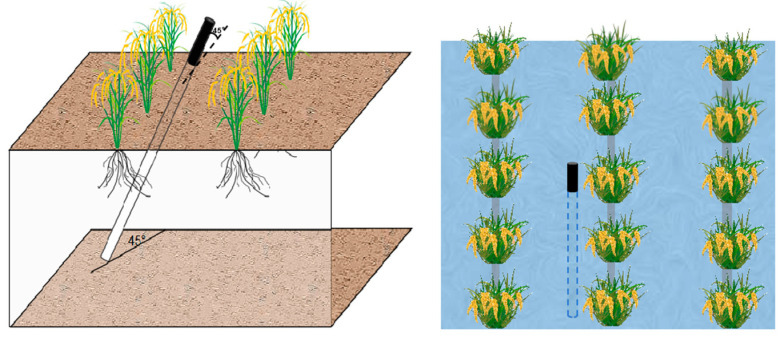
Microroot canal space layout.

Dynamic root monitoring began the grain-filling stage (80 DAE (days after emergence)) using the CI-600 in-situ root ecological monitoring system to observe roots at fixed points. The camera scanned soil layers at depths of 0–15 cm, 15–30 cm, 30–45 cm, and 45–60 cm at 5-day intervals. Root growth changes were continuously monitored without disrupting the growth process. Root tubes used in the experiment measured 1 m in length with an inner diameter of 6.9 mm, and each tube was installed at a 45° angle to the ground. The upper 20 cm of each tube was exposed above ground to attach the observation system’s positioning handle during monitoring. The exposed section was wrapped in black tape and sealed with a waterproof cap to prevent light penetration, protect root growth, and block dust or water from entering the tube, which could interfere with image collection (Figure 2).

(i) Acquisition of Root Morphological Parameters

Regular field root scans were performed to assess root standing stock. Image analysis generated data on key indicators, including total root length (RL, mm) and total root number (RN, no.). RL refers to the total length of fine roots (mm), while RN represents the total count of fine roots, both derived from image analysis.

(ii) Calculation of Root Architectural Parameters

Root architectural parameters were modeled using asymptotic equations to characterize the vertical distribution of root mass, root biomass, total root length, and root number, calculated as follows:

Y = 1 − *β*^D^

In the equation, D represents soil depth (cm); Y represents the proportion (0–1) of each morphological indicator from the surface to soil depth D; and *β* is the depth coefficient. The smaller the *β*, the closer the roots are distributed to the soil surface; the larger the *β*, the deeper the roots are distributed. In this study, values were calculated at a depth of D = 30 cm. The calculation was based on all data from three records during the observation period.

(iii) Daily Root Elongation Rate

The daily root elongation rate (RER; mm day^−1^) is calculated as the difference between the average total root length at time points t and t − 1, divided by the corresponding time interval:

$${\mathrm{RER}}_{t-1,1}=\frac{1}{N}\sum_{n=1}^{N}\frac{l_{n,t}-l_{n,t-1}}{P_{t-1,t}}$$

RER_t−1, t_ represents the root elongation rate (mm d^−1^) during the period between time points t − 1 and t; l_n,t−1_ and l_n,t_ are the root lengths at time points t − 1 and t, respectively; n denotes the root number; N is the total number of roots used for calculating the average daily RER; and P_t−1, t_ is the time interval between t − 1 and t.

RER_mean_ represents the average root elongation rate (RER) during the growth period. Dynamic root monitoring began 80 days after rice emergence (grain-filling stage) and continued for 20 days. Roots were observed at 5-day intervals, with 10 fine roots tracked per image. Each treatment was replicated three times.

#### 2.3.2. Root Biomass

At the flowering stage (75 DAE), two sampling points were chosen in each plot for each treatment. At each point, two rice plants with similar growth conditions were selected, and their aboveground parts were removed. Root samples were collected using the root drilling method (7 cm diameter, 10 cm height), with soil divided into 15 cm layers from 0 to 60 cm depth. Roots were separated from the soil cores, rinsed with tap water to remove impurities, and transported to the laboratory. The root samples from each soil layer were dried in an oven at 80 °C until reaching a constant weight and then weighed.

#### 2.3.3. Measurement of Root Activity

Root samples were collected twice during the grain-filling stage: mid-grain-filling (100 DAE) and late-grain-filling (120 DAE). Soil blocks containing roots (20 cm × 20 cm × 30 cm) were excavated from the field and immediately transferred into plastic buckets filled with tap water. Roots were gently rinsed under running water, and root activity was determined using the TTC method [31].

#### 2.3.4. Leaf Photosynthetic Characteristics

During the mid-grain-filling stage, ten plants with uniform growth were selected from each plot, and flag leaves were marked. A portable photosynthesis system (Yaxin-110, Beijing, China) was used to measure the photosynthetic parameters of the marked flag leaves between 10:00 and 12:00.

#### 2.3.5. Dry Matter Accumulation and N Uptake

At the maturity stage, two representative rice hills were collected from each plot. The samples were heat-treated at 105 °C for 30 min to deactivate enzymes, dried at 80 °C until reaching a constant weight, and weighed. The dried samples were ground, sieved through a 0.3 mm mesh, digested with concentrated H_2_SO_4_-H_2_O_2_, and the N content of each organ was analyzed using a FOSS-8400 semi-micro Kjeldahl N analyzer (Beijing, China).

#### 2.3.6. Superoxide Dismutase (SOD) and Malondialdehyde (MDA)

Rice plants with consistent growth and development were selected for leaf sampling the anthesis stage (75 DAE) and post-anthesis (95 DAE). About 0.1 g of rice leaves was ground in liquid N with 1 mL of buffer solution and then centrifuged at 8000× *g* for 10 min at 4 °C. The supernatant was used to measure superoxide dismutase (SOD) and malondialdehyde (MDA) levels. MDA content and enzyme activity were measured using the MDA assay kit (BC00025, Solarbio, Beijing, China), with absorbance determined at 532, 600, and 450 nm. SOD content and enzyme activity were determined using the SOD assay kit (BC0175, Solarbio, Beijing, China), with absorbance measured at 560 nm.

#### 2.3.7. Yield

Yield and yield components: Grain yield was estimated by selecting three 1 m^2^ areas from each plot, excluding edge effects. Effective panicles per square meter, grains per panicle, seed-setting rate, and 1000-grain weight were measured to calculate the yield.

### 2.4. Data Analysis

Data processing was conducted using Excel 2019. Variance analysis was carried out using SPSS 20.0 (SPSS, Chicago, IL, USA). The least significant difference (LSD) test was employed to compare the means, with differences considered significant at *p* < 0.05 (LSD 0.05). The means were further assessed using the LSD test. Graphs were created using Origin software (Version 9.0).

## 3. Results

### 3.1. Effects of Drip Irrigation Depth and N Forms on Rice Yield Formation and N Uptake

DI10-AN combined with ammonium N management significantly enhanced rice yield and N uptake, with no significant differences observed between years (Table 1). Over the two years, rice yield across treatments followed the order: DI10-AN > DI10-UN > DI0-AN > DI0-UN. Compared to other treatments, the DI10-AN treatment significantly increased 1000-grain weight by 4.26% to 10.22%, seed setting rate by 3.35% to 12.26%, yield by 13.32% to 46.31%, and N uptake by 14.37% to 52.88%.

### 3.2. Effects of N Forms and Drip Irrigation Depths on Leaf Enzyme Activity and Photosynthetic Characteristics of Rice

**Figure 3:**
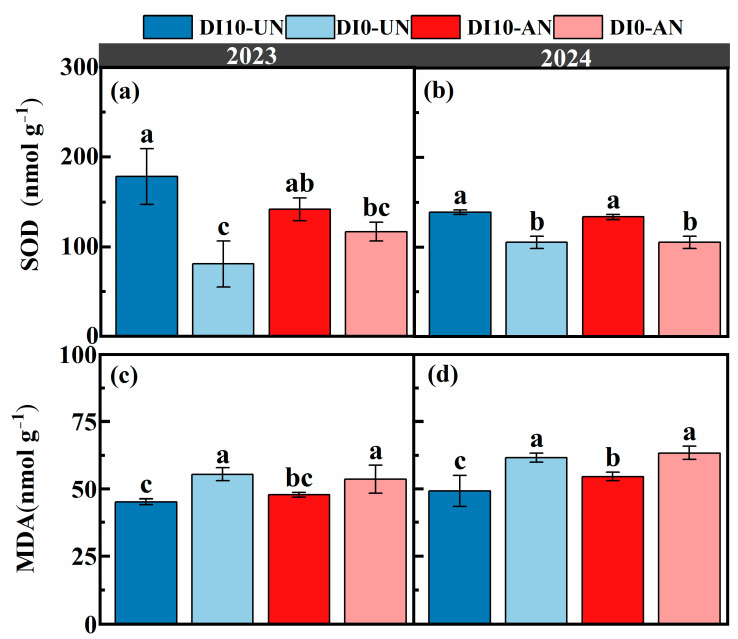
Effects of different drip irrigation depths and N forms on MDA and SOD in rice leaves during the grain-filling stage from 2023–2024. (**a**): SOD content during the grain-filling stage in 2023; (**b**): SOD content during the grain-filling stage in 2024; (**c**): MDA content during the grain-filling stage in 2023; (**d**): MDA content during the grain-filling stage in 2024. Different lowercase letters within the same column indicate significant differences between treatments (*p* < 0.05; LSD). DI0-UN: Conventional surface drip irrigation with urea N management; DI10-UN: Subsurface drip irrigation at 10 cm depth with urea N management; DI0-AN: Conventional surface drip irrigation with ammonium N management; DI10-AN: Subsurface drip irrigation at 10 cm depth with ammonium N management.

Over the two years, the SOD content in rice leaves followed the trend: DI10-UN > DI10-AN > D10-AN > D10-UN. Compared to surface drip irrigation (DI0-UN and DI0-AN), subsurface drip irrigation (DI10-UN and DI10-AN) significantly increased rice leaf superoxide dismutase (SOD) activity by 28.97% to 71.25%, while malondialdehyde (MDA) decreased by 14.7% to 16.98% (Figure 3). The net photosynthetic rate (Pn), transpiration rate (Tr), and stomatal conductance (Gs) were significantly higher under subsurface drip irrigation (DI10-UN and DI10-AN) treatments than those under surface drip irrigation (DI0-UN and DI0-AN), with the following order: DI10-AN > DI10-UN > DI0-AN > DI0-UN. No significant differences in Pn, Tr, and Gs were observed between DI0-UN and DI10-AN, as well as between DI0-AN and DI0-UN. Compared to other treatments, the DI10-AN treatment increased Pn by 4.22% to 18.34%, Tr by 8.35% to 28.28%, and Gs by 2.67% to 30.93% (Table 2).

### 3.3. Spatial Dynamic Distribution of Rice Root Morphology and Root Architecture (Root Biomass, Root Number, and Root Length)

#### 3.3.1. Root Biomass

**Figure 4:**
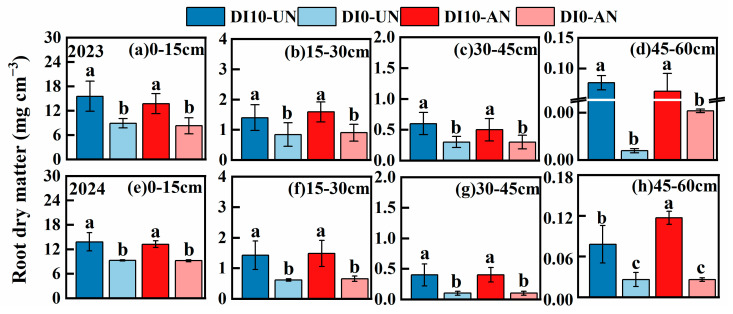
Root dry matter mass of rice at different soil depths during the grain-filling stage under various drip irrigation depths and N forms (2023–2024). Letters indicate significant differences among management practices within the same soil layer (*p* ≤ 0.05). Treatment abbreviations: DI0-UN: Conventional surface drip irrigation with urea N management; DI10-UN: Subsurface drip irrigation at 10 cm depth with urea N management; DI0-AN: Conventional surface drip irrigation with ammonium N management; DI10-AN: Subsurface drip irrigation at 10 cm depth with ammonium N management.

Compared to other treatments, the two-year average root biomass in the DI10-AN treatment increased significantly by 25.25% to 31.07%, while the root dry weight in the 0–30 cm soil layer increased by 23.19% to 28.36%. Significant differences in root dry matter were observed between the DI10-UN and DI10-AN and the DI0-UN and DI0-AN across different soil layers. In the 45–60 cm soil layer, root biomass increased significantly under DI10-UN and DI10-AN, with DI10-AN > DI10-UN (Figure 4).

#### 3.3.2. Root Length

**Figure 5:**
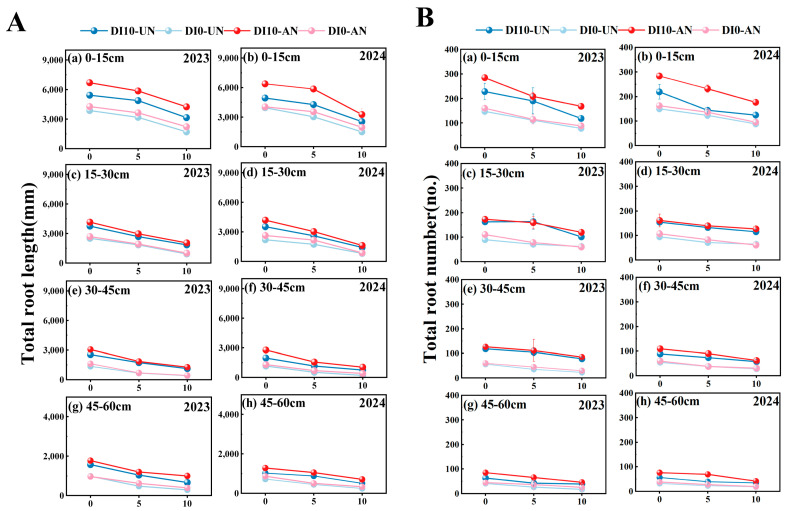
Dynamic changes in root length and root number decay across soil layers during the grain-filling stage under varying drip irrigation depths and N forms. (**A**): Total root length; (**B**): Total root number. Treatment abbreviations: DI0-UN: Conventional surface drip irrigation with urea N management; DI10-UN: Subsurface drip irrigation at 10 cm depth with urea N management; DI0-AN: Conventional surface drip irrigation with ammonium N management; DI10-AN: Subsurface drip irrigation at 10 cm depth with ammonium N management. The numbers 5, 10, and 15 denote the 5th, 10th, and 15th days of observation during the grain-filling stage.

DI10-AN significantly influenced root morphology at various soil depths, with a pronounced increase in root length in the 0–30 cm soil layer. Over the two years, at the initial observation stage (0 days), compared to other treatments, the root length (RL) in the DI10-AN treatment increased by 22.50% to 89.52%. From the start of grain-filling, root length declined progressively with crop development. The average root length decay rate followed the order: DI0-UN > DI0-AN > DI10-UN > DI10-AN, with significant differences among treatments. In the 30–60 cm soil layer, the decay rate was 14.67–38.69% higher than in the 0–30 cm layer (Figure 5A).

#### 3.3.3. Root Number

At the initial observation stage (0 days), compared to other treatments, the root number (RN) in the DI10-AN treatment increased by 20.57% to 101.61%. From the start of grain-filling, root numbers declined progressively with crop development. The average root number decay rate followed the order: DI0-UN > DI10-AN > DI10-UN > DI10-AN, showing significant differences among treatments. In the 30–60 cm soil layer, the decay rate was 24.15–37.05% higher than in the 0–30 cm layer (Figure 5B).

#### 3.3.4. Distribution of Root Architecture β Values

The root architecture was characterized by the magnitude of the *β* value derived from the asymptotic equation (Table 3). Different drip irrigation depths significantly influenced rice root architecture. Compared to DI0-UN, the *β* values of root dry matter (RDM), root length (RL), and root number (RN) under DI10-UN increased by 1.01–3.01%, 0.74–0.95%, and 0.52–0.84%, respectively. Similarly, compared to DI0-AN, the *β* values of RDM, RL, and RN under DI10-AN increased by 2.14–2.99%, 0.74–0.84%, and 0.40–0.45%, respectively. No significant differences were observed between N forms, but the overall trend followed: DI10-UN > DI10-AN; DI0-UN > DI0-AN.

### 3.4. Effects of Subsurface Drip Irrigation and N Forms on Daily RER and Ra in Rice

**Figure 6:**
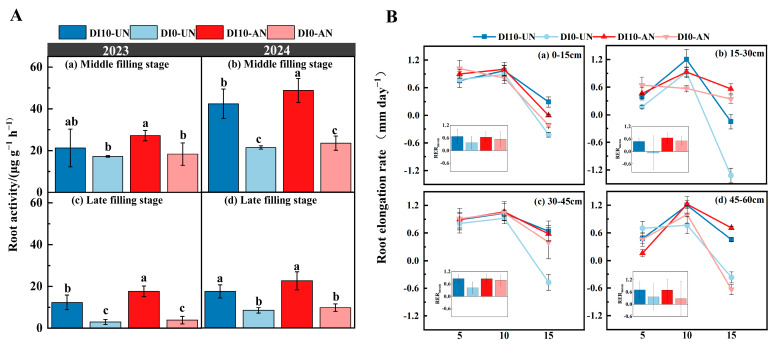
Changes in daily root elongation rate and root activity under different drip irrigation depths and N forms. (**A**): Root activity during the grain-filling stage from 2023–2024. (**B**): Dynamic changes in daily root elongation rate during the grain-filling stage in 2024. Treatment abbreviations: DI0-UN: Conventional surface drip irrigation with urea N management; DI10-UN: Subsurface drip irrigation at 10 cm depth with urea N management; DI0-AN: Conventional surface drip irrigation with ammonium N management; DI10-AN: Subsurface drip irrigation at 10 cm depth with ammonium N management. The numbers 5, 10, and 15 denote the 5th, 10th, and 15th days of observation. Different lowercase letters indicate significant differences between treatments (*p* < 0.05; LSD).

Among the two observation periods, DI10-AN exhibited the highest root activity in rice. Root activity across treatments decreased progressively as the growth period advanced. Over two years, root activity during the grain-filling stage followed the trend: DI10-AN > DI10-UN > DI0-AN > DI0-UN. Compared to other treatments, the average root vitality in the DI10-AN treatment increased by 23.24% to 133.72%. Among these, at the late grain-filling stage, root activity under DI10-AN and DI10-UN was significantly higher than under DI0-AN and DI0-UN. Compared to DI10-UN, root activity under DI10-AN increased by 28.78–42.1%, with no significant difference between the two. Compared to DI0-UN, root activity under DI10-UN increased significantly by 108.47–321.93%. Similarly, root activity under DI10-AN was 108.5–361.4% higher than under DI0-AN (Figure 6A).

The daily root elongation rate (RER) initially increased, peaking on the 10th day of observation before gradually decreasing. RER under subsurface drip irrigation treatments (DI10-AN and DI10-UN) showed no significant differences during the early and peak grain-filling stages. However, significant differences were observed during the late grain-filling stage between subsurface (DI10-AN and DI10-UN) and surface drip irrigation treatments (DI0-AN and DI0-UN), following the trend: DI10-AN > DI10-UN > DI0-UN > DI0-AN. Additionally, the average root elongation rate (RER_mean_) in different soil layers was higher under subsurface drip irrigation treatments (DI10-AN and DI10-UN) than under surface drip irrigation treatments (DI0-AN and DI0-UN) (Figure 6B).

### 3.5. Correlation Between Subsurface Drip Irrigation, Ammonium N, Root Morphological Parameters, and N Uptake

**Figure 7:**
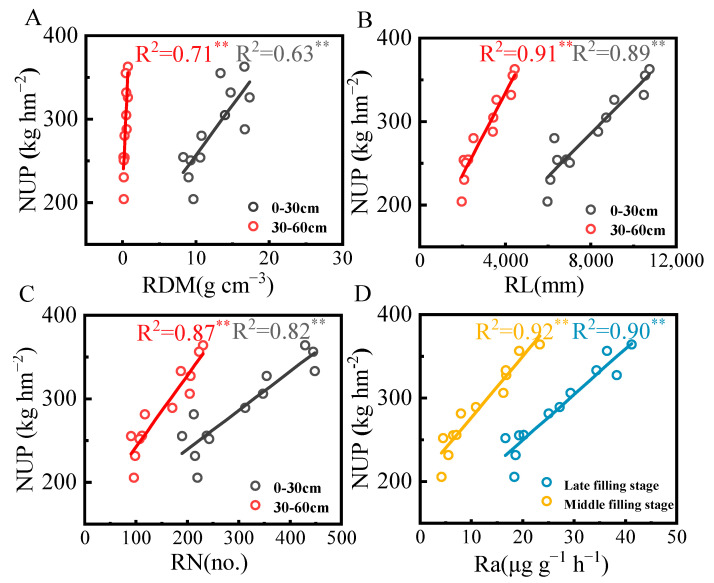
Correlation analysis between root morphology, root activity, and N uptake during the grain-filling stage (2023–2024). Note: ** indicates *p* < 0.01. (**A**): Correlation analysis between NUP and RDM. (**B**): Correlation analysis between NUP and RL. (**C**): Correlation analysis between NUP and RN. (**D**): Correlation analysis between NUP and Ra. Abbreviations: NUP: N uptake; RDM: Root dry matter; RL: Total root length; RN: Total root number; Ra: Root activity.

A significant correlation was observed between root morphological and physiological parameters and N uptake during the grain-filling stage in rice (Figure 7). N uptake in the aboveground parts was strongly positively correlated with root dry mass (RDM), root length (RL), root number (RN), and root activity (Ra), with RL and Ra showing the highest correlation coefficients. The data indicated that R^2^ values for root morphological indicators (RDM, RL, RN) in the 30–60 cm soil layer were higher than those in the 0–30 cm soil layer, suggesting that the distribution and physiological characteristics of deep roots were more strongly associated with N uptake in the aboveground parts.

### 3.6. Cluster Analysis and Correlation Analysis

**Figure 8:**
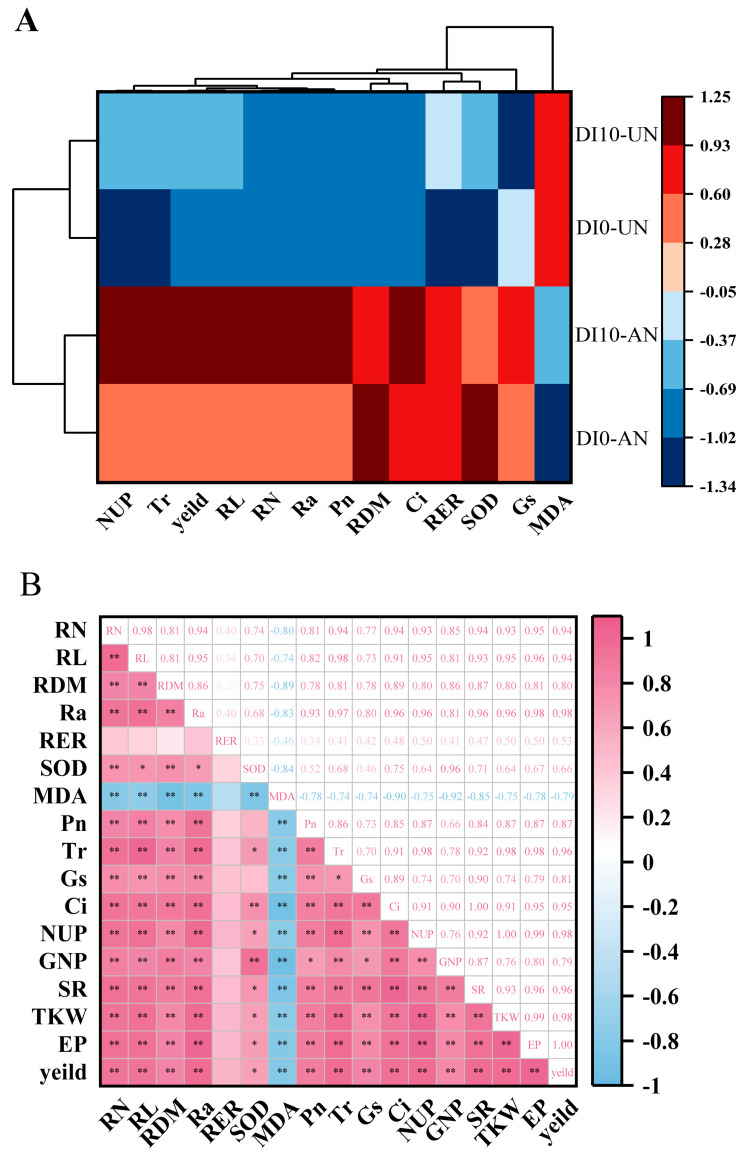
Cluster analysis and Pearson correlation analysis of various rice indicators (2023–2024). Data are presented as means (*n* = 3); * indicates significant correlation at *p* < 0.05; ** indicates significant correlation at *p* < 0.01. (**A**): the cluster analysis among indicators; (**B**): Pearson correlation analysis between indicators. Treatment abbreviations: DI0-UN: Conventional surface drip irrigation with urea N management; DI10-UN: Subsurface drip irrigation at 10 cm depth with urea N management; DI0-AN: Conventional surface drip irrigation with ammonium N management; DI10-AN: Subsurface drip irrigation at 10 cm depth with ammonium N management. Indicator abbreviations: RDM: Root dry matter; RL: Total root length; RN: Total root number; Ra: Root activity; RER: Root elongation rate; NUP: N uptake; GNP: Grain number per panicle; SR: Seed setting rate; TKW: 1000-kernel weight; EP: Efficient panicle.

A simple cluster analysis of various rice indicators was performed to develop a robust evaluation system (Figure 8A). The results revealed that, based on the CA model, the four treatments could be grouped into two distinct categories. DI10-AN and DI10-UN exhibited a high degree of similarity in indicator expression, reflecting comparable effects on root growth, photosynthetic production capacity, and leaf senescence, forming a clear contrast with DI0-AN and DI0-UN. Among the indicators, NUP, Tr, yield, RL, RN, Ra, and Pn showed strong correlations (Figure 8A), further validated by Pearson correlation analysis (Figure 8B). Pearson correlation analysis indicated that RL, RN, and Ra were significantly positively correlated with Tr, Ci, NUP, TKW, SR, EP, and yield (r > 0.90). SOD showed significant positive correlations with RDM and GNP, with correlation coefficients of 0.89 and 0.92, respectively. TKW exhibited extremely strong positive correlations with NUP and Tr, with correlation coefficients of 1 and 0.98, respectively. Yield was strongly positively correlated with Ra, NUP, and TKW, with a correlation coefficient of 0.98. These findings indicate that, while some overlap exists among the indicators, each is essential and irreplaceable in reflecting the factors that influence yield.

## 4. Discussion

### 4.1. Subsurface Drip Irrigation Combined with Ammonium N Effectively Maintains Root Activity in the Late Growth Stage of Drip-Irrigated Rice

Root activity is a comprehensive indicator of the ability of plant roots to absorb, transport, and utilize water and nutrients, as well as their adaptability to soil environmental conditions. Enhanced root activity promotes root growth, thereby increasing nutrient absorption [32,33,34]. This study demonstrated that subsurface drip irrigation (DI10-AN and DI10-UN) significantly increased rice root activity by 25.8–47.9% (Figure 6A). This effect may be attributed to the deep burial of drip irrigation tape, which facilitated the vertical movement of surface water and nutrients, enabling deeper roots to access these resources and promoting their growth and development [35,36]. Lateral root tips exhibit the highest metabolic and respiration rates, which are closely associated with high root turnover [37,38]. As roots age, N absorption efficiency declines, leading plants to enhance root activity to maintain normal respiration and metabolism for nutrient acquisition [39]. Consistent with these findings, our field experiments revealed that subsurface drip irrigation (DI10-UN and DI10-AN) effectively maintained root elongation rate and root activity during the late grain-filling stage while reducing root decay rates compared to surface drip irrigation (DI0-UN and DI0-AN) (Figure 4, Figure 5 and Figure 6).

The survival and growth of fine rice roots in deeper soil layers are critical for maintaining root activity, ensuring normal plant growth, and achieving high yields during the late growth stages [40]. Although rice naturally develops a shallow root system, high-frequency irrigation concentrates water and nutrients in the shallow soil layers, which further promotes the concentration of roots in these layers, limiting their growth and downward rooting. Since N availability typically declines with increasing soil depth [41], subsurface drip irrigation promotes the downward redistribution of water and nutrients, encouraging plants to develop a deep root system capable of stable growth and elongation in deep soil. This study demonstrated that the root elongation rate in the 45–60 cm soil layer under subsurface drip irrigation (DI10-UN and DI10-AN) was significantly higher during the late growth stage compared to surface drip irrigation (DI0-UN and DI0-AN) (Figure 6B). This result is because the nutrient-rich environment near the absorptive fine roots under subsurface drip irrigation better supports the normal physiological activity of the root system than surface drip irrigation [42,43]. These findings support the hypothesis that an appropriate spatial distribution of nutrients can maintain root activity in rice during the late growth stage, thereby promoting deeper root distribution, increasing total root length and root biomass in deeper soil layers, and mitigating shallow root distribution and early root senescence (Figure 4 and Figure 5).

### 4.2. Subsurface Drip Irrigation Combined with Ammonium N Promotes Root Growth and Proper Distribution in Drip-Irrigated Rice

Root plasticity, encompassing morphology, structure, and function, represents a key response to soil nutrient characteristics [44,45]. Grasses demonstrate (*Poaceae*) greater root morphological plasticity than legumes (*Fabaceae*), with localized high-nutrient concentrations stimulating root growth [5,23]. This study revealed that deep N application (DI10-UN and DI10-AN), compared to surface drip irrigation, significantly enhanced root growth and optimized root architecture in rice (Figure 3, Figure 4 and Figure 5, Table 3). Specifically, DI10-AN promoted substantial root growth in the 0–30 cm soil layer, suggesting that the spatial heterogeneity of nutrients and water formed by DI10-AN benefits shallow root growth (Figure 3, Figure 4 and Figure 5, Table 3). This growth reflects an adaptation to heterogeneous environments, where plants occupy nutrient-rich zones to maximize resource acquisition [19,46]. The *β* value of root architecture under subsurface drip irrigation (DI10-UN and DI10-AN) was significantly higher than under surface drip irrigation (DI0-UN and DI0-AN) (Table 3), indicating that DI10-AN not only promotes shallow root growth but also enhances deep root growth, encouraging greater root penetration [19]. This difference can be attributed to two main factors: (1) More than 80% of rice roots are concentrated in the shallow (0–30 cm) soil layer, and ammonium N, easily adsorbed by soil anion colloids, exhibits limited mobility in the soil. (2) Subsurface drip irrigation delivers water and nutrients directly near the crop roots, reducing N diffusion distance, minimizing volatilization and leaching risks [47], and creating nutrient-enriched, well-moistened zones that enhance N uptake. This study demonstrates that DI10-AN establishes an effective N “reservoir” throughout the rice growing season, ensuring sustained nutrient availability and promoting root growth [48].

Changes in root architecture growth strategies enhance the utilization of plant-available nutrients. The dynamic interplay between root plasticity and morphology allows plants to adapt to changing environmental conditions. Alterations in root architecture influence nutrient and water absorption, which, in turn, affect root growth and branching [6,49]. Deep fertilization targets the primary root growth layer in rice, improving spatial alignment between root growth and N distribution, thereby optimizing root architecture [50]. Accordingly, this study suggests that DI10-AN not only influences root morphological traits but also ensures spatial alignment between nutrient distribution and root growth.

### 4.3. Subsurface Drip Irrigation Combined with Ammonium N Promotes N Accumulation and Yield Formation in Drip-Irrigated Rice

The rice root system anchors the plant, absorbs nutrients and water, and synthesizes hormones such as amino acids and cytokinins, maintaining a balance between morphology and function with the aboveground parts. This study aimed to evaluate the responses of root growth, photosynthesis, N uptake, and yield to shallow subsurface drip irrigation and different N forms. In this experiment, N uptake in the aboveground parts was significantly positively correlated with RDM, RL, RN, and Ra (Figure 7 and Figure 8B). This suggests that a robust root architecture and strong activity can significantly enhance N uptake, and rice yield is closely linked to N accumulation at maturity. Higher N accumulation leads to greater rice yield. Additionally, compared to the shallow soil layer (0–30 cm), root morphological parameters (RDM, RL, RN) in the deeper soil layer (30–60 cm) exhibited a stronger correlation with N uptake (Figure 5). This is because deep root growth expands the plant’s capacity to access N in the soil, which is particularly critical for N uptake during the maturity stage.

Optimizing root architecture is essential for improving plant adaptability to adverse environmental conditions [51]. The evaluation system established through cluster analysis indicates that root growth regulation under the DI10-AN is better suited for plant adaptation in heterogeneous environments (Figure 8A). Enhancing photosynthetic capacity is critical for increasing crop yield [52,53]. In this study, subsurface drip irrigation treatments (DI10-UN and DI10-AN, with DI0-UN > DI0-AN) effectively maintained photosynthetic and transpiration rates during the grain-filling stage. Furthermore, RDM, RL, RN, and Ra were significantly positively correlated with Pn and Tr. These findings suggest that subsurface drip irrigation strengthens the root system, enhances net photosynthetic and transpiration rates during the grain-filling stage, and facilitates N uptake and yield improvement (Table 2, Figure 8B). This aligns with previous studies showing that drought-tolerant rice varieties with well-developed and deep root systems enhance transpiration and photosynthesis, ultimately increasing dry matter accumulation [9]. Improving photosynthetic efficiency during the late growth stages is particularly important for crop yield formation. This study also demonstrated that the DI10-AN reduced malondialdehyde (MDA) content and increased superoxide dismutase (SOD) content in leaves, alleviating the negative impacts of drought stress on plant growth during the late growth stage. Consequently, it helped maintain high photosynthetic productivity [54].

Root growth is strongly linked to aboveground N uptake and yield formation (Figure 8B), as roots and aboveground parts interact closely. Vigorous aboveground growth ensures the transport of sufficient carbohydrates to the roots, maintaining and enhancing root function. In turn, active roots supply adequate nutrients and water to the aboveground parts, promoting growth and increasing N uptake [55,56].

Thus, optimizing drip irrigation depth and N form in intensive crop production has the potential to enhance crop yield by improving the synchronization between nutrient supply and root uptake [43]. This study demonstrated that DI10-AN significantly increased rice yield, offering valuable insights for drip-irrigated field crops. It is important to note that this study focused on subsurface drip irrigation at a depth of 10 cm. However, the optimal application depth may vary depending on regional factors such as crop type, climate, irrigation methods, and the ratio of basal to topdressing fertilizer.

Roots are essential for regulating plant functions, nutrient uptake, and aboveground development. This study employed minirhizotron technology to monitor dynamic changes in live root length, root number, and root elongation rate in situ, offering new perspectives on managing root dynamics through drip irrigation depth and N forms in rice production under arid conditions. While most studies focus on perennial tree roots, research on crop root dynamics in agricultural systems is relatively scarce. This study provides valuable insights into root development strategies for annual crops that are highly responsive to soil environmental changes. The optimal burial depth for subsurface drip irrigation varies depending on factors such as soil texture, crop type, and irrigation requirements. Future research should focus on how subsurface drip irrigation depth affects water use efficiency, crop yield, and soil moisture distribution under varying soil types and crop conditions, aiming to refine irrigation parameters and enhance system performance.

## 5. Conclusions

The morphological strategies of rice roots demonstrated significant responses to variations in drip irrigation depth and N forms. This study introduced an innovative drip irrigation technique that integrates DI10-AN into rice cultivation. The key findings are as follows: (1) The DI10-AN notably enhanced root proliferation within the 0–30 cm soil layer, optimized the spatial distribution of root architecture, and encouraged deeper root penetration. (2) The DI10-AN maintained root activity during the grain-filling stage, reduced rates of root decay, and postponed root senescence. (3) The DI10-AN mitigated water and N stress in leaves during the later stages of growth by lowering malondialdehyde (MDA) levels and increasing superoxide dismutase (SOD) activity, which supported high photosynthetic productivity and boosted yield.

This study introduces a novel subsurface drip irrigation technique (10 cm depth) combined with ammonium (300 kg ha^−1^) for rice cultivation, offering a fresh perspective for drip-irrigated rice production in Xinjiang, China. This technique presents a novel fertilization method that promotes root growth in the drip irrigation system and enhances crop productivity. In this study, the selected subsurface drip irrigation depth is 10 cm, which is somewhat limited. Therefore, determining the optimal application depth for subsurface drip irrigation remains an area for future investigation and exploration.

## Figures and Tables

**Table 1:** Effects of drip irrigation and N management at different locations on rice yield component.

Year	Treatment	Efficient Panicle 10^4^ ha^−1^	Seed Setting Rate (%)	Grain Number per Panicle	1000-Kernel Weight (g)	Yield (t ha^−1^)	N Uptake (kg ha^−1^)
2023	DI10-UN	360.82 ± 2.2.9 a	76.79 ± 3.74 a	86.57 ± 1.03 a	32.17 ± 0.71 ab	7.72 ± 0.59 ab	149.38 ± 6.54 b
	DI0-UN	367.32 ± 14.99 a	76.35 ± 1.11 a	78.93 ± 0.19 c	31.01 ± 1.56 ab	6.86 ± 0.44 b	108.28 ± 16.80 c
	DI10-AN	376.31 ± 10.49 a	81.15 ± 5.63 a	84.22 ± 0.67 b	33.08 ± 0.18 a	8.51 ± 0.59 a	179.59 ± 5.23 a
	DI0-AN	336.33 ± 15.31 b	80.73 ± 7.12 a	84.17 ± 0.88 b	30.04 ± 0.78 b	6.87 ± 0.73 b	121.77 ± 6.39 c
2024	DI10-UN	365.82 ± 6.87 a	79.09 ± 2.56 ab	82.66 ± 0.82 a	31.88 ± 0.36 a	7.62 ± 0.27 b	156.40 ± 12.73 ab
	DI0-UN	320.84 ± 17.68 b	72.21 ± 1.21 b	71.89 ± 0.73 c	30.12 ± 1.49 b	5.02 ± 0.45 c	120.47 ± 8.27 c
	DI10-AN	369.82 ± 8.26 a	85.42 ± 5.10 a	84.84 ± 0.80 a	33.11 ± 0.53 a	8.88 ± 0.75 a	170.14 ± 11.20 a
	DI0-AN	336.83 ± 5.27 b	73.36 ± 0.99 b	77.02 ± 0.72 b	30.01 ± 0.71 b	5.72 ± 0.44 c	139.36 ± 9.58 bc

Different lowercase letters within the same column indicate significant differences between treatments during a single growing season (*p* < 0.05; LSD). Treatment abbreviations: DI0-UN: Conventional surface drip irrigation with urea N management; DI10-UN: Subsurface drip irrigation at 10 cm depth with urea N management; DI0-AN: Conventional surface drip irrigation with ammonium N management; DI10-AN: Subsurface drip irrigation at 10 cm depth with ammonium N management.

**Table 2:** Effects of different drip irrigation depths and N forms on photosynthetic characteristic parameters at the grain-filling stage.

Year	Treatment	Pn (mmol CO_2_ m^−2^s^−1^)	Tr (mmol H_2_ Om^−2^s^−1^)	Gs (mmol m^−2^s^−1^)	Ci (μmol CO_2_ m^−2^s^−1^)
2023	DI10-UN	7.19 ± 0.54 a	5.26 ± 0.22 b	124.97 ± 12.71 ab	183.7 ± 17.76 b
	DI0-UN	6.47 ± 0.78 b	4.32 ± 0.13 c	124.2 ± 24.88 ab	130.72 ± 31.00 c
	DI10-AN	7.41 ± 0.35 a	6.1 ± 0.13 a	136.63 ± 10.99 a	217.12 ± 11.76 a
	DI0-AN	6.55 ± 0.22 b	5.10 ± 0.13 b	105.7 ± 5.91 b	122.97 ± 21.50 c
2024	DI10-UN	6.75 ± 1.09 ab	5.67 ± 0.11 a	120.58 ± 10.78 a	147.68 ± 24.38 a
	DI0-UN	5.82 ± 0.54 b	5.16 ± 0.09 b	104.27 ± 7.82 bc	111.5 ± 37.31 a
	DI10-AN	7.11 ± 1.00 a	5.71 ± 0.45 a	115.75 ± 11.25 ab	130.87 ± 14.51 a
	DI0-AN	5.92 ± 0.74 b	4.95 ± 0.09 b	96.47 ± 7.25 c	107.68 ± 37.59 a

Different lowercase letters within the same column indicate significant differences between treatments during a single growing season (*p* < 0.05; LSD). Treatment abbreviations: DI0-UN: Conventional surface drip irrigation with urea N management; DI10-UN: Subsurface drip irrigation at 10 cm depth with urea N management; DI0-AN: Conventional surface drip irrigation with ammonium N management; DI10-AN: Subsurface drip irrigation at 10 cm depth with ammonium N management. Indicator abbreviations: Pn: Net photosynthetic rate; Tr: Transpiration rate; Gs: Gas conductance; Ci: Intercellular CO_2_ concentration.

**Table 3:** Effects of different drip irrigation depths and N forms on the distribution of root architecture *β* values in rice.

Year	Treatment	RDM *β*	RL *β*	RN *β*
2023	DI10-UN	0.900 ± 0.001 a	0.958 ± 0.002 a	0.963 ± 0.002 a
	DI0-UN	0.891 ± 0.004 b	0.951 ± 0.004 b	0.955 ± 0.004 b
	DI10-AN	0.908 ± 0.005 a	0.958 ± 0.001 a	0.963 ± 0.001 a
	DI0-AN	0.889 ± 0.004 b	0.951 ± 0.001 b	0.959 ± 0.001 ab
2024	DI10-UN	0.890 ± 0.009 b	0.954 ± 0.002 a	0.959 ± 0.001 a
	DI0-UN	0.864 ± 0.010 d	0.945 ± 0.006 b	0.954 ± 0.002 b
	DI10-AN	0.896 ± 0.006 a	0.955 ± 0.003 a	0.959 ± 0.003 a
	DI0-AN	0.870 ± 0.005 c	0.947 ± 0.005 b	0.955 ± 0.00 b

Different lowercase letters within the same column indicate significant differences between treatments during a single growing season (*p* < 0.05; LSD). Treatment abbreviations: DI0-UN: Conventional surface drip irrigation with urea N management; DI10-UN: Subsurface drip irrigation at 10 cm depth with urea N management; DI0-AN: Conventional surface drip irrigation with ammonium N management; DI10-AN: Subsurface drip irrigation at 10 cm depth with ammonium N management. Indicator abbreviations: RDM: Root dry matter; RL: Total root length; RN: Total root number.

## Data Availability

The data presented in this study are available on request from the corresponding authors.

## Abbreviations

The following abbreviations are used in this manuscript:

RL	Total root length
RDM	Root dry matter
Ra	Root activity
RN	Total root number
RER	Root elongation rate
SOD	Superoxide Dismutase
MDA	Malondialdehyde
Pn	Net photosynthetic rate
Tr	Transpiration rate
Gs	Gas conductance
Ci	Intercellular CO_2_ concentration
NUP	N uptake
GNP	Grain number per panicle
SR	Seed setting rate
TKW	1000-kernel weight
EP	Efficient panicle
